# Metrics used in quality improvement publications addressing environmental sustainability in healthcare: A scoping review protocol

**DOI:** 10.1371/journal.pone.0309417

**Published:** 2024-08-28

**Authors:** Colin Sue-Chue-Lam, Sezgi Yanikomeroglu, Doulia Hamad, Brian Wong, Karen Born

**Affiliations:** 1 Institute of Health Policy, Management & Evaluation, Dalla Lana School of Public Health, University of Toronto, Toronto, ON, Canada; 2 Centre for Quality Improvement and Patient Safety, Temerty Faculty of Medicine, University of Toronto, Toronto, ON, Canada; 3 Division of General Surgery, Department of Surgery, University of Toronto, Toronto, ON, Canada; 4 Department of Medicine, University of Toronto, Toronto, ON, Canada; 5 Sunnybrook Health Sciences Centre, Toronto, ON, Canada; Addis Ababa University, ETHIOPIA

## Abstract

Quality improvement approaches are increasingly being used to address the problem of healthcare’s climate and ecological impact. While sustainability is increasingly recognized as a domain of quality, consensus is lacking on the most appropriate measures and metrics for those looking to reduce ecological impacts through quality improvement initiatives. We propose a scoping review to summarize approaches for selecting and quantifying ecological impacts in the published quality improvement literature. We will search multiple electronic databases (MEDLINE, EMBASE, CINAHL, and Scopus) from 2000 onwards, to identify published quality improvement initiatives in the human healthcare setting intended to address ecological impact with at least one quantitative measure of ecological impact, such as kilograms of carbon dioxide equivalent greenhouse gas. Two independent reviewers working in parallel will screen studies for inclusion and abstract study data, including publication, study, and ecological impact characteristics. Charted data will be synthesized narratively as well as with descriptive tables, figures, and summary statistics. In doing so, we will map areas of relative focus as well as gaps in the measurement of ecological impact across quality improvement initiatives. This map can in turn be used to raise awareness of ecological impacts requiring broader consideration, encouraging holistic and clinically relevant approaches to measuring ecological impact in future quality improvement work.

## Introduction

### Rationale

The healthcare sector ought to promote human health and wellbeing. Yet, healthcare has a significant global ecological impact with harmful downstream effects on health.

[1–4] Greenhouse gas emissions (GHG) from the US health care system climbed to 8.5% of total US GHG in 2018. Pollution from the US health care sector including GHG, PM2.5, and ozone exacted a toll of 388,000 disability-adjusted life-years in 2018 alone [3]. Global health care sector pollution was associated with the loss of 4 million DALYs in 2020 [5]. These serious health harms are preventable and pose an opportunity for the health sector to demonstrate leadership in the sustainability transformation.

Healthcare providers and organizations are increasingly turning to quality improvement (QI) as the science of systematic and continuous improvement to healthcare processes in attempts to mitigate the ecological impacts of healthcare [6, 7]. Existing efforts, while still early, focus mostly on climate impacts [8]. However, well-intentioned interventions to mitigate climate change may trade off against negative impacts on other earth system processes [9]. Intravenous anesthetics can sometimes be substituted for inhaled anesthetics with high global warming potential, but the common intravenous anesthetic propofol is often improperly disposed of, having toxic effects on aquatic life that cannot be ignored [10]. In this way, the planetary boundaries literature makes clear that a truly sustainable healthcare sector must attend to its relationship with an array of earth system processes [9].

Unfortunately, we know little at present about how QI initiatives have identified and measured the full range of ecological impacts–to the climate and beyond [11]. Indirect evidence from the healthcare life cycle analysis literature suggests that non-climate ecological impacts may be neglected in QI initiatives. The HealthcareLCA living database has catalogued studies of environmental impact of healthcare, noting that 99% (151/152) of studies catalogued climate impacts but only 55% (83/152) of studies evaluated any other impact category (such as acidification potential, particulate matter formation, and freshwater ecotoxicity potential, among others) [12]. While suggestive of a relative neglect in QI initiatives, the evidence from the LCA literature falls short of supporting any strong claim about prevailing approaches to measuring ecological impact in the sustainable QI literature.

Given that robust and meaningful measures are a cornerstone of QI, and furthermore that unrecognized tradeoffs may stymie efforts to build sustainable health systems, we must better understand how existing QI efforts to address environmental sustainability in healthcare have attempted to identify and measure ecological impacts. The primary aim of the proposed scoping review is thus to investigate this question and determine the gaps and areas of emphasis across this emerging body of literature. By mapping existing approaches to measuring ecological impact in the sustainable QI literature, we can suggest ways to leverage strengths, address weaknesses and inform future clinical and scholarly work.

## Methods

This protocol was prepared using guidance for the development of scoping review protocols [13]. We will use the scoping review extension for the Preferred Reporting Items for Systematic Reviews and Meta-Analyses (PRISMA) to guide reporting of the completed review [14]. Any amendments to the study protocol will be described in the methods section of the final publication.

We have chosen a scoping review approach over a systematic review approach because it suits our research aims, which relate to mapping methodological approaches and gaps in a new, diverse body of literature rather than arriving at an unbiased and precise effect estimate [15, 16].

### Eligibility criteria

Clinically-led quality improvement (QI) or clinical innovation efforts taking place in all human healthcare delivery settings (acute care, post-acute care, ambulatory care) will be eligible for inclusion. We define clinical innovation efforts as ideas, services, or treatments used in a clinical setting that display benefits over current practices [17, 18]. Interventional studies will be included. The concept of interest is the exploration of metrics used to measure environmental impact in QI and clinical innovation efforts in the healthcare setting. Studies attempting to address environmental sustainability and that provide a quantitative measurement of ecological impact will be included. Measurement of ecological impact may be done directly or indirectly. We define direct metrics of environmental impact to include metrics such as kilograms of carbon dioxide equivalent greenhouse gas or water utilization. We define indirect metrics of ecological impact as clinical metrics with environmental impacts, such as the number of medication vials avoided, or the weight of plastics captured for recycling. We expect to find a broad range of metrics, beyond those identified in key papers retrieved from our initial search.

Modelling studies, reviews, case reports, editorials, letters, commentaries, and other publications that do not report any primary data or present new analyses of existing data will be excluded. Studies describing QI or clinical innovation efforts taking place outside of the clinical activity in the healthcare setting (i.e., physical plant, exclusively operations or engineering) will be excluded. Studies that are not peer-reviewed or published in languages other than English will also be excluded.

### Search strategy

We have designed a comprehensive search strategy in collaboration with experienced information specialists and a quality improvement research synthesis expert. An initial search was completed on MEDLINE to retrieve relevant articles. Subject headings and keywords used in titles and abstracts of the articles of interest were used to create a full search strategy. The terms capturing QI and clinical innovation efforts included variations of the terms “quality improvement” and “clinical innovation”. The terms capturing ecological impact included “carbon”, “waste”, “overuse”, “environment”, “sustainability” and variations of these terms related to both direct and indirect ecological impacts. The healthcare setting was captured using variations of the term “healthcare”. The strategy was validated through the retrieval of several key studies, determined *a priori* to meet inclusion criteria. The full MEDLINE search strategy is detailed in Appendix 1 (“S1 File”). The full search strategy will be translated into all included databases.

Included search results were limited to those published in English and on or after the year 2000.

We limited results to those published on or after the year 2000 because this was the year the influential *To Err is Human* report was published and QI emerged internationally as a field of study [19]. Furthermore, the environmental impacts of healthcare have not been widely published until the last two decades [6, 20, 21].

### Information sources

MEDLINE, EMBASE, CINAHL, and Scopus will be searched during August 2023 with database-specific search strategies for peer-reviewed published literature. The MESH terms selected for the search in MEDLINE were adapted to corresponding headings in each database.

### Grey literature

Patient Safety Network (PSNet), Healthcare Without Harm, Cascades, UK Sustainability in Quality Improvement (SusQI), Choosing Wisely Canada National Meeting Abstract Books, and Institute for Healthcare Improvement (IHI) will be searched using keyword combinations derived from our main literature search. These organizations and websites were selected based on group discussion and input from subject matter experts that they are subject domain leaders in quality improvement and sustainability.

### Data management

Covidence will be used to manage duplicates and screen titles, abstracts, and full-texts. Piloted Microsoft Excel spreadsheets will be used to chart the extracted data.

### Selection process

Two independent reviewers will conduct two-stage screening in duplicate using piloted screening forms at each stage of screening. The first stage is abstract and title review, with eligibility confirmed by two independent reviewers. Any disagreements will be resolved through discussion and consensus, involving a third reviewer if consensus cannot be achieved. The second screening stage will be conducted on full text articles, again in duplicate with disagreements resolved through discussion, consensus, and similarly involving a third reviewer if consensus cannot be achieved.

### Data collection process

Data will be extracted in duplicate by two independent reviewers using a pilot-tested data abstraction form. The pilot-tested abstraction form will be run for a random sample of ~30 articles by reviewers to calibrate internal consistency among reviewers. Resulting changes will be made before continuing extraction for the remaining articles. Any disagreements will be resolved through discussion and consensus, involving a third reviewer if necessary. As the two reviewers proceed, the data charting form will be revised iteratively as needed.

### Data items

Data items are described in Table 1. We expect that many studies will include multiple outcomes. In these cases, we will extract data for all reported outcomes.

**Table 1:** Data abstraction table. Items planned for abstraction from the fulltext results.

Publication characteristics	Study characteristics	Measurement characteristics	Quality improvement intervention characteristics
Year of publication	Country of origin	Metric	QI intervention (e.g. audit and feedback, clinician education)
Study duration	Setting (e.g. ward, operating room/acute care, primary care)	Direct or indirect environmental impact	
First author	Health discipline (e.g. medicine, nursing) and subdiscipline (e.g. general surgery, general internal medicine)	Absolute, relative, or both scales	
Journal	Study design		
	Trial funding source		

### Data synthesis

We will present data narratively. We will also present a measurement framework to organize the data items described in Table 1, a figure to illustrate publications over time, and a diagram to illustrate the overlap between potential metrics of environmental impact and the metrics of environmental impact utilized in our dataset. Potential metrics of ecological impact will be identified from key references in life cycle assessment literature [8, 18].

### Institutional review board statement

Not required in the setting of a review of published literature.

## Limitations

While our results will represent a comprehensive review of sustainable QI and clinical innovation initiatives, not all QI initiatives are published [22]. While published QI initiatives are more likely to be successful than unpublished QI initiatives, our aim is not to establish effectiveness, thereby minimizing the impact of this particular limitation of our sample. Nonetheless, to mitigate this limitation we have designed a comprehensive grey literature search strategy to capture initiatives outside the peer-reviewed literature.

All human activities have ecological impacts. We have thus limited our inclusion to studies with an explicit intent to address environmental sustainability. QI initiatives and clinical innovations not intending to address environmental sustainability will nonetheless have ecological consequences or co-benefits, and our study would not capture these. As a result of this exclusion, our study will be best positioned to provide insights for clinicians, policymakers, and scholars interested in understanding the state of the developing field of sustainable QI and clinical innovation science.

We have limited our search to studies published in English, leaving our results best positioned to describe the landscape of sustainable QI initiatives and clinical innovations in countries where English is dominant, or where researchers are able to publish their work in English.

## Conclusions

Healthcare providers and systems serve to protect, promote and preserve human health. As such, they have a direct role and responsibility to curb climate impact, reduce ecologic harms and promote sustainability. However, measuring these changes can be challenging. The results of the proposed scoping review will address this critical knowledge gap by providing a systematic overview of how QI and clinical innovations targeted at environmental sustainability have operationalized this broad aim.

Our work will raise awareness of healthcare’s broad ecological footprint ‐ inclusive of but not limited to its effects on climate change ‐ among clinicians, QI researchers, and organizations. For clinicians, we will identify prior QI activities that might be used as a guide to future on-the-ground sustainability-focused QI efforts and encourage them to take a broad view on what constitutes an ecological impact. For QI scholars and researchers, our results will characterize the scale of existing sustainable QI efforts, identifying which settings and clinical domains have seen greatest activity and QI priority-setting. For organizations interested in embedding sustainability into QI plans and priorities, this work will offer an approach to measuring impact and outcomes. The ultimate impact of this work will be to support guidance and recommendations to those undertaking the urgent task of advancing sustainability in healthcare.

## Supporting information

S1 File MEDLINE search strategy. (DOCX)

S1 Checklist PRISMA-P (Preferred Reporting Items for Systematic review and Meta-Analysis Protocols) 2015 checklist: Recommended items to address in a systematic review protocol*. (DOC)
