# Supplementary material for: Metrics used in quality improvement publications addressing environmental sustainability in healthcare: A scoping review protocol
Source: PLoS One. 2024 Aug 28;19(8):e0309417. doi: 10.1371/journal.pone.0309417 (PMC11356433; doi:10.1371/journal.pone.0309417)
Supplement: S1 File — (DOCX) [file pone.0309417.s001.docx]

**S1 Figure. MEDLINE search strategy.**

Ovid MEDLINE: Epub Ahead of Print, In-Process & Other Non-Indexed Citations, Ovid MEDLINE® Daily and Ovid MEDLINE® <1946-Present>

| Database: MEDLINE  Date: 14/08/2023 | Search | Results |
| --- | --- | --- |
| #1 | exp Quality Improvement/ or exp Total Quality Management/ or exp Quality Assurance, Healthcare/ | 381211 |
| #2 | exp Quality Indicators, Healthcare/ | 24994 |
| #3 | (clinic* adj2 innovat*).tw,kf. | 2222 |
| #4 | exp Pilot Projects/ | 148576 |
| #5 | exp Efficiency, Organizational/ | 22437 |
| #6 | exp Organizational Innovation/ | 27805 |
| #7 | exp Organizational Objectives/ | 21119 |
| #8 | exp Medical Audit/ | 17657 |
| #9 | exp "Cost Savings"/ | 12732 |
| #10 | service evaluation.tw,kf. | 1984 |
| #11 | healthcare efficiency.tw,kf. | 141 |
| #12 | waste management program*.tw,kf. | 102 |
| #13 | component analysis.tw,kf. | 57591 |
| #14 | life cycle assessment.tw,kf. | 4248 |
| #15 | 1 or 2 or 3 or 4 or 5 or 6 or 7 or 8 or 9 or 10 or 11 or 12 or 13 or 14 | 669429 |
| #16 | exp "Delivery of Healthcare"/ | 1232787 |
| #17 | Operating Rooms/ | 15948 |
| #18 | exp Hospitals/ | 319679 |
| #19 | exp Critical Care/ | 66940 |
| #20 | exp Perioperative Care/ | 158799 |
| #21 | exp Ambulatory Care/ | 56540 |
| #22 | exp Anesthesia/ | 206422 |
| #23 | exp Anesthesiology/ | 33353 |
| #24 | exp Emergency Medical Services/ or exp Emergency Service, Hospital/ | 170007 |
| #25 | exp Intensive Care Units/ | 106400 |
| #26 | exp Academic Medical Centers/ | 102379 |
| #27 | (clinic* or medic* or doctor* or physician* or anaesthe* or anesthe* or surg* or nurs* or hospital* or care or diagnostic imaging).kf. | 1033481 |
| #28 | health facilities/ or hospital units/ or intensive care units/ or hospitals/ or rehabilitation centers/ | 207181 |
| #29 | surgical procedures, operative/ | 56864 |
| #30 | medicine/ or "allergy and immunology"/ or anesthesiology/ or clinical medicine/ or community medicine/ or dermatology/ or emergency medicine/ or pediatric emergency medicine/ or general practice/ or hospital medicine/ or integrative medicine/ or internal medicine/ or neurology/ or palliative medicine/ or pathology/ or pediatrics/ or perioperative medicine/ or "physical and rehabilitation medicine"/ or psychiatry/ or radiology/ or reproductive medicine/ or specialties, surgical/ or sports medicine/ or telemedicine/ or nursing/ or nursing, practical/ or nutritional sciences/ or optometry/ or pharmacology/ or pharmacy/ or psychology, medical/ or toxicology/ | 532547 |
| #31 | (hospital* or operating room or surg*).tw. | 3614482 |
| #32 | 16 or 17 or 18 or 19 or 20 or 21 or 22 or 23 or 24 or 25 or 26 or 27 or 28 or 29 or 30 or 31 | 5965717 |
| #33 | exp Climate Change/ or exp Climate/ | 185283 |
| #34 | Greenhouse Effect/ | 6159 |
| #35 | Greenhouse Gases/ | 2429 |
| #36 | exp Carbon/ or exp Carbon Dioxide/ or exp Carbon Footprint/ or exp Carbon Cycle/ | 307115 |
| #37 | Conservation of Natural Resources/ or Conservation of Energy Resources/ or Conservation of Water Resources/ | 51676 |
| #38 | Water Supply/ or exp Water Pollution/ | 61808 |
| #39 | Waste Products/ or exp Waste Management/ or exp Medical Waste/ | 103711 |
| #40 | Global Warming/ | 4436 |
| #41 | exp Ozone Depletion/ | 70 |
| #42 | Ocean Acidification/ | 51 |
| #43 | exp Recycling/ | 6544 |
| #44 | exp Disposable Equipment/ | 5288 |
| #45 | (climate adj2 crisis).tw,kf. | 661 |
| #46 | (planetary adj2 health).tw,kf. | 1032 |
| #47 | (greenhouse adj3 (gas* or effect*)).tw,kf. | 15695 |
| #48 | life cycle assessment.tw,kf. | 4248 |
| #49 | emission*.tw,kf. | 304306 |
| #50 | (carbon adj2 (dioxide or footprint or foot print or emi* or cycl*)).tw,kf. | 79353 |
| #51 | recycl*.tw,kf. | 68688 |
| #52 | pollution.tw,kf. | 124805 |
| #53 | ((eco* or environ*) adj2 (responsibility or friendly)).tw,kf. | 36357 |
| #54 | ((environ* or ecolog*) adj2 (sustainab* or foot print or footprint or impact or effect* or results* or cost* or burden* or resource* or conserv* or benefi* or damag* or harm* or consequence* or releas*)).tw,kf. | 87896 |
| #55 | ((medical or hospital or surg* or sterile or operating room or lab* or clinic* or test* or instrument* or resource*) adj2 (wast* or garbage or overuse or unused or stewardship)).tw,kf. | 8646 |
| #56 | waste*.kf | 40405 |
| #57 | sustainability.kf. | 8660 |
| #58 | green.kf. | 22403 |
| #59 | (particulate adj2 nitrogen).tw,kf. | 336 |
| #60 | toxic particle*.tw,kf. | 113 |
| #61 | Air Pollution/ | 40195 |
| #62 | Fossil Fuels/ | 3026 |
| #63 | fossil fuel*.tw,kf. | 7647 |
| #64 | 33 or 34 or 35 or 36 or 37 or 38 or 39 or 40 or 41 or 42 or 43 or 44 or 45 or 46 or 47 or 49 or 50 or 51 or 52 or 53 or 54 or 55 or 56 or 57 or 58 or 59 or 60 or 61 or 62 or 63 | 1256874 |
| #65 | 15 and 32 and 64 | 3382 |
| #66 | limit 65 to (english language and yr="2000 -Current") | 2806 |
